# Reasons for not using ecstasy: a qualitative study of non-users, ex-light users and ex-moderate users

**DOI:** 10.1186/1471-2458-12-353

**Published:** 2012-05-14

**Authors:** Maria Angé licade Castro Comis, Ana Regina Noto

**Affiliations:** 1 Department of Psychobiology, UNIFESP (Universidade Federal de São Paulo), Rua Botucatu, 862 1o andar - Vila Clementino, São Paulo, SP CEP 04023-062, Brazil

## Abstract

**Background:**

Although ecstasy is often consumed in the electronic music scene, not everyone with the opportunity to use it chooses to do so. The objective of this study was to understand the reasons for non-use or the cessation of use, which could provide information for public health interventions.

**Methods:**

A qualitative reference method was used. Our “snowball” sample group consisted of 53 people who were split into three subgroups: non-users (NU, n = 23), ex-light users (EX-L, n = 12) and ex-moderate users (EX-M, n = 18). Individual, semi-structured interviews were conducted, transcribed and subjected to content analysis with the aid of NVivo8.

**Results:**

Adverse health effects and personal values were given as reasons for non-use in the three groups. Non-users (NU) and ex-light users (EX-L) provided reasons that included fear of possible effects as well as moral, family and religious objections. Ex-moderate users (EX-M) cited reasons related to health complications and concomitant withdrawal from the electronic music scene. However, most of the ex-moderate users did not rule out the possibility of future use.

**Conclusions:**

Potential effects and undesirable consequences appear to guide the decisions within the different groups. Prevention might target these motivations. Individuals who have used ecstasy indicate that social and environmental factors are the most important factors.

## Background

Ecstasy (MDMA, 3,4-methylenedioxymethamphetamine) has been used by young adults in every continent and the prevalence rates vary depending on country, population and point in time. In the USA, the National Household Survey on Drug Use (NHSDA) reported that the lifetime prevalence of ecstasy use increased from 2% in 1995 to 13% in 2001 [1]. In Brazil, a national survey of 12,711 university students indicated a lifetime prevalence of 7.5% [2].

The primary sites of MDMA use have been nightclubs, electronic music festivals and rave parties [3]. Young adults attend these events featuring electronic music and colorful decor [4,5]. These venues usually have areas where people can dance, talk and rest. Participants are looking for fun and they usually go in groups. Historians of the electronic scene observed that the use of ecstasy is closely related to parties. Participants reported that dancing under the effect of ecstasy is more pleasant and they state that the main effect of the drug is to induce empathy [6]. Studies on participants in the electronic music scene in Australia [7], the Netherlands [8] and Taiwan [9] have suggested a lifetime prevalence of ecstasy use between 20% and 77%.

Most investigative studies acknowledge the importance of social context and electronic music in the initiation and continued use of ecstasy. Vervaeke et al. found that initiation of use among 106 ecstasy users in Amsterdam was mainly due to peer pressure and curiosity, while continuation was more related to socializing with other users [10].

However, some studies have indicated that the reasons given for ecstasy use cessation can guide preventive and therapeutic strategies [11]. Studies with ex-users have focused on this perspective. Peters, Kok & Schaalma observed that ex-users reportedly ceased use because of acquisition of new responsibilities, loss of interest, acute and severe effects of experimentation and decreased participation in dance events [12]. Other studies, including one conducted in Brazil, corroborated the relationship between the interruption or reduction of use and lifestyle changes as well as withdrawal from the electronic music scene [12-14]. Health issues also appear to influence cessation of use. In the UK, one cessation study identified two distinct groups of motivation. One group gave circumstantial reasons, such as the decision to stop going out at night, while the other group was motivated by mental health concerns, including symptoms of depression, anxiety and paranoia. In Germany, cessation of use was attributed to the dual fears of compromising occupational performance and negative health consequences [15]. A focus group study of university students highlighted a variety of reasons: negative experiences (bad trips), health concerns, financial problems, tolerance and dependence, loss of interest, negative observations of other people who use the drug and the fear of legal consequences [15].

Research suggested the need for more detailed studies with ex-users to deepen the understanding of cessation factors. A qualitative study in the USA found that 8.6% of a sample of 115 individuals discontinued ecstasy use due to negative experiences, erection difficulties and paranoia. Subjects stated that the risks had begun to outweigh the benefits associated with consumption [16].

Future studies should account for different age groups and different use contexts [11,17]. Most studies have been conducted in North America and Europe; thus, new studies on ex-users need to clarify cessation patterns, especially in other cultural contexts, including Latin America.

Another information gap involves the segment of the public who has never used ecstasy or who did not advance past experimental use. Although 1.9% of Brazilian college students reported recent use within the last 30 days, 92.5% never used ecstasy, while 4.4% used it in their lifetime but not within the last year [2]. An understanding of the reasons individuals resist MDMA use when the drug is available, such as in the electronic music scene, might inform and promote discussion regarding preventive measures [11].

Peters, Kok & Schaalma stated the fear of its effects and health risks, including dependence, motivated people not to use MDMA even when it was accessible [12]. However, this study did not elucidate whether the opportunity to use was only characterized by the fact that the individuals had been somewhere where the drug is used or whether they had been offered the drug [10]. Even greater knowledge gaps exist in experimental users, as studies on this social group have not been performed.

The objective of this study was to understand the beliefs of different groups who choose not to use ecstasy even when presented with the opportunity. Subjects are categorized into three groups: ex-moderate users, ex-light users and non-users, in the most populous city in South America, Sao Paulo.

## Methods

Qualitative research procedures were used. According to Peters and Kok, such procedures are useful for studies on the discontinuation of ecstasy use and deepen understanding of the phenomenon [11].

To familiarize the researcher with the context and application of research methodology, we completed observational studies in eight parties, five interviews with key informants and a pilot study with two interviewees.

### Participants

*Intentional Criterion Sampling* was used [18]. The sample group consisted of individuals over 18 years of age without cognitive impairment or evident psychiatric disorder that could bias the content of interview. The specific terms that categorize user type follow Sterk et al (2007) [19]:

a) **Non-user (NU):** Participants had never consumed ecstasy despite having at least one practical opportunity. An opportunity was considered to be any context with explicit drug availability and/or drug offer.

b) **Ex-light user (EX-L):** Participants had consumed ecstasy from 1 to 5 times in their lifetime, but at least 12 months had passed since their last use [19,20].

c) **Ex-moderate user (EX-M)**: Participants had consumed ecstasy more than 5 times during their lifetime, but at least 12 months had passed since last use. This cutoff point was based on qualitative studies that defined specific drug users as people who have used ecstasy 5 or more times [19,20].

The sample group was composed of 53 volunteers (23 NU, 12 EX-L and 18 EX-M), between 2009 and 2010. The search was performed with the "snowball" technique, which creates a chain of potential new cases from a first interviewee. Respondents belonged to different professions, including lawyers, psychologists, musicians, engineers and sales representatives. They were approached in different contexts, ranging from academic, artistic, medical, festive and other areas and appointed by key informants, such as party promoters, DJs and regular rave attendees with special knowledge about the study population [17]. This technique provides an advantageous access to a population that otherwise cannot be reached [20]. Eight participant observations were conducted in contexts with a high likelihood of usage, including parties and electronic music festivals. The parties were open to the general public and the author learned about them through the Internet. The observation of participants was not used for recruiting; it was intended to acquaint the interviewer with the interviewees’ familiar context. Those observations also facilitated both the author’s insertion in the electronic scene and her future contact with the interviewees.

Some difficulties were encountered in composing the NU group. These setbacks likely occurred because social networks searches intended to find non-users with genuine opportunity to use the drug often lead to actual users. Users rarely mentioned or maintained relationships with people who were not using, especially those users who had not used for more than 12 months.

To increase the number of user profiles and to meet all the criteria proposed for the initial investigation, a maximum of three participants per chain were pursued. Data collection for the study was completed when the interviews reached the "point of theoretical saturation"; i.e., the interviews became redundant regarding reasons for non-use or cessation [18,21].

### Interviews

Invitations for individual volunteers were arranged by the people who referred them. If a subject accepted the invitation, the subject received further detailed information via a phone call and/or email. Before the interview, the terms of free and informed consent were presented. A single researcher conducted all of the semi-structured, individual interviews in neutral venues with guaranteed anonymity. The interviews were carried out in closed rooms and the interviewer advised the volunteers that they could stop whenever they wanted, although none voluntarily ended the interview. The interviews were conducted in Portuguese, the language spoken in Brazil. These procedures were approved by the Ethics Committee of the UNIFESP (ECP 1740/08).

Semi-structured interviews followed a script that covered topics including reasons for non-use or cessation of ecstasy use, socio-demographic characteristics, history of ecstasy use and life changes (change in city, civil status, relationships, responsibilities, etc.). The interviews were 45 to 60 minutes long.

### Processing and analysis of the contents

The interviews were transcribed verbatim and each was given a corresponding alphanumeric code composed of the following sequence: group acronym (NU, EX-L and EX-M), gender and age. The principal researcher compared the literal audio transcription, in its entirety, to the original audio. Preliminary analysis and review were performed with the participation of other two senior researchers. Analysis evaluated the pilot study, sample size, choice of analysis categories and the result coding. The senior researchers made suggestions to improve the pilot interview and facilitated data triangulation after collection. Informed by their qualitative research experience, they evaluated coding and categorization.

The content of each transcript was processed with the aid of NVivo8 software, which facilitated the topic grouping, interview codification and thematic categorization [22]. Categories were analyzed using the content analysis technique, a common analytical method for qualitative data. In this technique, the material is encoded and organized into categories, which transform the raw data into a possible representation of the contents [22]. The following recurring, relevant themes guided the result description: contexts of and/or opportunity for use, reasons for non-use and/or cessation of use, protective factors (family and religion), health hazards, risk perception, use perspectives, dependence and lifestyle changes.

## Results

The three groups had similar socio-demographic characteristics. The median age was 26 years in the NU and EX-L groups, while the median age in the EX-M group was 28 years. Men and women were uniformly distributed among the groups. The socio-economic level for the majority of the sample was middle class. The majority of respondents had completed higher education. Most of the individuals were single and active in the labor market in variety of occupations and only four subjects were unemployed.

Almost all of the respondents reported lifetime use of alcohol and tobacco. The majority of respondents from the NU group and almost all of the EX-L and EX-M subjects reported prior use of other illicit drugs. The latter group reported a wider range of previously used drugs (including LSD and ketamine).

### Context of use and/or opportunities for use

The three groups cited the electronic music scene as the main context in which they used or had the opportunity to use ecstasy. However, the EX-M and EX-L groups reported that they used the drug at other types of parties, such as nightclubs, university parties, *micaretas* (a popular party that is similar to Brazilian Carnival but held on different dates) and even rodeos (a popular party composed of shows and horse or cattle riding). The NU reported having had the opportunity to experiment with the drug in many different parties. This diversity of contexts suggested that the opportunity to use was associated with party environments in general and was not limited to the electronic scene.

One participant (EX-M) reported that his last usage of ecstasy was in prison; he had been detained for selling ecstasy tablets. At least five members of the EX-M group reported heavier drug use while abroad, mainly in London. They reported continued less frequent use after returning to Brazil. 

"A rave is very colorful. The electronic sound is really cool. You can dance there, not look at anyone and stay on your trip. It’s an amusement park of drugs. Everybody's on drugs, but everyone’s happy, nobody’s worried about family or money problems, nobody." (Ex-M, f, 31)"

"I used it once on the beach. It was a reggae show…it was bad because I wanted to keep walking without stopping." (Ex-l, f, 31)"

"I was with a guy at a micareta party and his friend offered it to me… imagine if I had taken it at this crowded party?" (Nus, f, 27)"

### Protective factors — family and religion

The majority of the NU group stated that family values influenced their decision to refuse ecstasy consumption. In the EX-L group, half of the respondents mentioned family beliefs as a contributing cessation factor. However, the EX-M group reported that family values had almost no influence on their decision to halt ecstasy consumption, instead citing other factors. The main reasons cited by those respondents who reported a familial influence included the fear of disappointing family and/or failing to be accepted by family. 

"Their opinion is important to me. I ask myself what my mother would think if I had been using it and such." (Nus, f, 24)"

"I could have used more often, but I didn't want to. My parents taught and guided me to be against drugs…I used more out of curiosity." (Ex-l, m, 24)"

When asked about religious influence, half of the NU group reported that religion influenced them to not use the drug. In the EX-L group, half reported that religious aspects and family principles contributed to cessation. In the EX-M group, religious factors were not cited as important for drug consumption cessation. 

"The religious values that I had made me think a little of the consequences, not of the 'I am going to hell' kind, but in the sense of thinking about afterwards." (Nus, f, 24)"

"When I took ecstasy, I didn't want to go to church because I thought I had sinned, so I thought a lot about it and today I don't think I sinned, but I also don't want to use any more". (Ex-l ,m, 28)"

“I've always been mystical. I always liked esotericism, and always followed several lines within esotericism since I was fifteen years old, but this did not affect my use of ecstasy nor when I stopped." (Ex-M, f, 28)"

### Adverse effects

In the EX-L group, the predominant reasons for cessation included physical, psychological and cognitive complications that were attributed to the experimental use of ecstasy. Physical complications included bad hangovers, eye tremors, vomiting, bruxism, cracked teeth and sore mouth. The EX-L group stated that they did not enjoy the effects caused by drug ingestion. Psychological complications included symptoms of depression and anxiety, bad trips and a feeling of intense fear. The main cognitive complication mentioned in the EX-L group was attention deficits while under the influence or shortly after use of the drug. 

"I was very shaken, not shaken in a way that is right. It didn't give me the blast I expected it to. I was not tripping. " (Ex-L, m, 26)"

"I dunno. I felt very hot, sweated a lot, feeling a fever as well…and vomited.”(Ex-L, f, 28)"

In the EX-M group, the predominant reasons for cessation were complications perceived as the effects of long-term use. Physical problems included bruxism, weight loss, muscle pain, worsening hangovers and weakness. The psychological complications in the EX-M group and EX-L group were similar, except for the feelings of intense fear. Among the cognitive issues, memory problems and lack of concentration were most commonly cited.

Two participants, with heavy use for a year or more, reported memory loss even after they stopped using ecstasy. One respondent reported that she developed bruxism after taking ecstasy. Even after 12 months without the drug, she was still grinding her teeth while she slept. Some ex-moderate users reported weight loss during their use of the drug.

The EX-M reported different histories of ecstasy use. The duration of use ranged from 2 to 10 years and the frequency of use ranged from four times a week to once a month. 

"I felt like I had been hit by a truck. I spent the entire next day sleeping, completely worn out, although it was pretty cool. Somehow I've been forgoing this." (Ex-M, f, 24)"

"I came back without a brain. What I feel today is lost memory, medium and long-term. Things happened before London and I don’t remember anything." (Ex-M, m, 26)"

### Risk perception

All three groups perceived ecstasy to be a dangerous drug that might lead to social and physical problems. The vast majority of the NU Group, which had never used ecstasy despite opportunities to do so, perceived ecstasy as an accessible drug for people who like to experiment with substances or get numb for fun. However, they reported believing that ecstasy use encourages risky behaviors including, but not limited to, unprotected sex and driving under the influence of the drug. This group attributed their refusal to use ecstasy to the fears of loss of control, dependence and physical illness (e.g., nausea and vomiting). They mentioned the fear of more severe acute effects, such as aggravation of an existing heart problem and/or seizures.

"The minute I put any drug in my body, I do not know what it is and have no control over its quality." (Nus, f, 28)"

The EX-L group reported the belief that drug use can be harmful because the composition of each tablet is unknown. The EX-M group reported that ecstasy is a drug that cannot be used frequently because it causes substantial physical and emotional weakness; thus, it should only be used as a part of a party ritual. 

"It put me at risk for sure…I took it in combination with alcohol, so it was a pretty big risk." (Ex-M, m, 26)"

"It lasts longer than other drugs and makes you do some things you probably never would otherwise." (Ex-L, f, 31)"

### Dependence

The NU and EX-L groups acknowledged the possibility of ecstasy dependence but argued that context is a very important factor predicting continued use. However, the interviewees from the EX-M group reported that the consumption of ecstasy does not create chemical dependence. They associated drug use with context, in the sense that their desire to consume was elevated in certain social contexts. 

"At least the people I know are not dependent on ecstasy. They are dependent on other things but make use of ecstasy, mainly related to the environment they are in ". (Nusam28)"

"It didn't make sense to go to a rave without taking a pill, nor to take a pill without going to a rave, because the whole deal was about mixing things". (Ex-M, f, 25)"

### Changes in lifestyle and expected future use

The majority of respondents in the EX-M group cited life changes as the reason for ecstasy cessation. The main reported changes were professional matters (such as increased responsibilities), a change of city and/or country, the start or end of relationships or personal growth. Respondents additionally perceived the behavior of other people under the influence of ecstasy to be "ugly or ridiculous". They described this factor as another reason for cessation of use. 

"My responsibilities have increased; the last time I used [the next day] it compromised all my work in the lab, so I stopped going to parties…I was really dumb the day after." (Ex-M, f, 25)"

"I got bored of seeing the behavior the moment a person gets high on the drug, you know? I got to see people kissing. I began to see even that as an ugly thing. I do not want and would not want other people to see me that way." (Ex-M, f, 27)"

Most of the interviewees in the NU group reported that they would not experiment with ecstasy if they were in a drug use context. One interviewee stated that she would use ecstasy drug if she did not have marijuana, her drug of choice. The majority of the interviewees in the EX-L group reported they would not use ecstasy again. 

"Nowadays, I don't think there is a situation where I would use it, it's not prejudice, but because I just don't think I'd want to…I don't think it had much effect on me when I used it." (Ex-L, m, 28)"

"It's a drug that never brought me any benefit, the propaganda was misleading for me. I think it’s a useless drug". (Ex-L, m, 41)"

Interestingly, the majority of the EX-M group reported that they would use ecstasy again, even after 12 months without use. Those who gave this response reported that most of them no longer visit the context of use. When questioned about events or situations in which they would use drugs again, these respondents reported probable use should they be in locations where they had previously used ecstasy. Only four volunteers reported that they did not intend to use ecstasy again because of drug associated debilitation and other complications. 

"Maybe if I were at some rave again. I don’t completely exclude the possibility of reuse." (Ex-M, f, 25)"

"I would like to because I find the effect of the [bala] (a slang for ecstasy) very pleasurable. Today I am aware of the associated risks, so I guess that makes me more careful than back then". (Ex-M, f, 28)"

## Discussion

The results of this study indicated that the reasons for rejecting ecstasy varied according to interviewee drug perception and experience level. Non-users (NU) and ex-light users (EX-L) more often gave reasons related to moral, family and religious principles as well as to the fear of adverse effects. However, people who stopped after a period of chronic use (EX-M) tended to attribute cessation to physical, psychological and social complications. They also cited maturity and professional concerns that motivated a parallel withdrawal from the context of use. Despite ecstasy cessation for more than 12 months, these respondents reported the possibility of future use in situations of opportunity, such as the electronic scene.

One of the aspects that deserve to be considered is our difficulty to find people who had not experienced ecstasy after having an opportunity to use it; it was easier to find ecstasy users than to find ex-users (abstinent for 12 months). The average age of our sample group was slightly higher than the average found in most ecstasy studies. The prevalence of tobacco use was also higher than in other Brazilian youth samples. These characteristics deserve attention in the interpretation of results [13,15,23]. The interviewees' reports did not necessarily include all of the reasons for rejecting ecstasy because the report content was limited to what the respondents were aware of and could verbalize in an interview.

In the non-users group, several subjects reported the use of other drugs, both licit and illicit, which differed from studies with other populations that included higher proportions of absolute non-users [20,24]. This difference was possibly related to the study’s inclusion criteria, which were aimed at people who had faced genuine opportunities to use ecstasy, particularly in the electronic scene. The active nightlife characteristic might have differentiated the sample. The characteristic could also be interpreted as a study strength because it highlighted people most vulnerable to drug use. To ensure the specificity of the data to ecstasy, care was taken during the interviews to distinguish it from other drugs [12,25].

The NU group reported using a smaller variety of illicit drugs compared to the wide range of drugs previously consumed by people in the EX-M group. This finding corroborated Scholey et al. who noted a significant difference in the range of previous illicit drugs consumption by ecstasy users compared to non-users [26].

The results of this study suggested that ecstasy use was associated with parties in general. The possible development of dependence appeared to be connected to the context of use. The sample reported ecstasy use at different types of parties, which demonstrated diversity in the contexts of use. However, all contexts shared common elements, such as music, dance, social interaction and having fun. The effects of the drug can explain the pairing of ecstasy with partying. However, context was a greater risk factor for chronic use. Classical conditioning, for example, is a theory that can be exploited to understand the use of ecstasy in specific contexts [27].

Cognitive and behavioral models of drug dependence support the idea that environmental events trigger drug use. The reaction to cues associated with the drug might represent an important risk factor for the transition from recreational use to addiction [28]. Given that behavior is learned through classical conditioning and associated with social learning, the strength of the association depends on the frequency of joint occurrence of the stimuli. If context and stimuli do not appear together, the conditioning is reduced or remission occurs [29]. This logic contributed to our understanding of reported cessation that ex-moderate users who had moved away from the electronic scene. Alternatively, it also suggests that withdrawal from the electronic scene is an important choice that supports people who wish to stop using.

Even when they had experienced complications that provoked the cessation of ecstasy use, ex-moderate users reported that they would resume drug use if they started attending raves and similar parties again. Because all participants met the study criterion of 12 months without ecstasy use, the perception of unpleasant symptoms might have diminished. The individual might consequently be more open to new episodes of use. Falck et al. studied 304 ex-ecstasy users (18 to 30 years old) who answered questions about resuming use of the drug. The responses were: ‘no’, 8.3%; ‘probably not’, 16.2%; ‘did not know’, 15.8%; ‘probably’, 37.3%; and ‘definitely yes’, 22.4% [30]. This prevalence suggests that ex-users of ecstasy tend to want to use the drug again. Behavioral models of dependence should be taken into account in association with the context of use.

Singer and Schensul report that some individuals lost control of their ecstasy consumption. The users reported several unsuccessful cessation strategies. These results differ from those in our study. Therefore, more studies should focus on individuals who try to stop using the drug [16].

The contexts and social networks in which young adults are immersed are also involved in the use of ecstasy. Vervaeke et al. found that it is common for individuals who continue using ecstasy to remain close to other users, maintaining their social networks focused on consumption [10]. Consumption is restricted to certain subcultures and compatible with collective representations of pleasure seeking, feelings of belonging, and transgression [31].

Lifestyle changes were noted as reasons for cessation of ecstasy use. Withdrawal from the context of use often followed increasing responsibilities within the professional sphere, fear of diminishing efficiency, changing personal relationships and loss of interest in the drug [10,32]. This latter aspect could be related to a tolerance that developed through use and/or to a decrease in the quality of the pills ingested [33]. However, cessation could also be part of a transitional phenomenon during young adulthood that reflects lifestyle changes and biopsychosocial alterations that increase the likelihood of withdrawal from the contexts of use and other practices [14].

Although ex-moderate users less frequently cited family and religious beliefs, the opposite trend occurred in the NU and EX-L groups. Non-users and ex-light users highlighted religious and family values as relevant factors for their ecstasy use decisions. Several studies have emphasized the important influence of moral and/or religious principles, coupled with parental influence, in substance use decisions [20,24,34-36]. Moral values seem to be significant factors in the early stages of drug use avoidance. However, for those still using ecstasy, such values are given different meanings that do not seem to represent a barrier to use.

The literature states that loss of interest and legal ramifications motivate the cessation of consumption. Our sample did not mention these reasons [12,24,34]. Some of the most common explanations were related to health complications, such as cognitive, physical and psychological problems. A longitudinal study of ecstasy users demonstrated that heavy users had more memory difficulties than newer users [37,38]. Among psychological problems, other studies stated that symptoms such as depression, anxiety and paranoia occur frequently [39-41]. In the UK, 56% of a sample associated cessation with mental health problems [39]. In a study by Falck et al., individuals who had consumed ecstasy on more than 50 occasions showed a higher incidence of severe depressive symptoms than people who had consumed up to 10 times [42].

Physical health statements from ex-moderate users in this study were consistent with the international literature. The most commonly reported physical problems in other studies were tremors, weight loss and bruxism [32,40]. Parrott found that positive use effects diminished, while negative symptoms increased over time among newer ecstasy users [38].

Individual vulnerability is another aspect relevant to cessation. According to Vervaeke et al., some individuals have a greater vulnerability to drug components and may experience worse sensations during ecstasy consumption. Although respondents from the EX-L group did not make this observation, it could explain their discontinuation of ecstasy consumption [10]. Given this hypothesis, the EX-L group could be more vulnerable to the negative effects of ecstasy compared to the EX-M group.

However, the NU and EX-L groups shared similar fears of the negative effects of ecstasy. These results corroborate studies that cited fear as a major reason for the cessation of ecstasy use, specifically fear of psychosocial compromises, loss of control, dependence and health damage [10,24,34]. The two groups additionally had similar views on the role of family values and religious matters. Thus, several factors potentially influence the rejection of ecstasy or the decision to not escalate beyond experimental use. Some of these factors could have practical prevention application, such as the appreciation that ecstasy use is not required to enjoy a party. In this sense, fun without ecstasy is a possible promotional message to dissuade drug abuse.

A review by Peters et al. explored social determinants of the ecstasy consumption and found that many have positive expectations associated with use of the drug [43]. It is important that these aspects are demystified so that young adults can obtain reliable information about the unwanted effects of the drug. One should bear in mind that the negative effects of the drug might be associated with contaminated pills. A Brazilian chromatography study of 25 different ecstasy batches found MDMA variations from 30.9 to 92.7 mg. This information could strategically discourage non-users from experimentation and warn current users [44]. Because users cannot test the pills in Brazilian parties, it is difficult to know actual pill composition.

Most respondents reported that they obtain information about the positive and negative effects of the drug through the Internet and/or users. This response suggested that information enhances perception of risk. Young individuals are searching for further information of the drug, possibly due to media coverage of deaths associated with ecstasy consumption. Ex-users also reported access to harm reduction (HR) information, which has been a widely used strategy for damage reduction during and after the consumption of ecstasy [15,39,45].

A harm reduction (HR) strategy should be considered because of the association observed in this study between the use of ecstasy in party environments and the intention of ex-users to resume drug usage in these contexts. Various strategies for HR are discussed in the literature. In some contexts, the vendors show users how to use (e.g., ingesting water, food, visiting the *chill-out*, etc.) [34]. Non-governmental organizations (NGOs) that practice HR at electronic music festivals assess the purity of tablets and assist users having bad trips. These interventions *in loco* aim to reduce risky behaviors among ecstasy users and ensure a minimal amount of safety for people who opt to take ecstasy [34,46]. The lifestyles of ex-light users and ex-moderate users, combined with complications resulting from ecstasy use, underscore the importance of intervention *in loco*. These users usually do not utilize the health care system, which makes it difficult to understand consumption patterns, complications and spontaneous remission [15,33,34,36].

## Conclusions

Study results provided information on the different forms of ecstasy use intervention. Strategies aimed at non-users and ex-light users should be different from ones developed for persistent users. Dissemination of information about the effects and/or adverse consequences of use seem to be an important strategy for prevention. Discussion of life goals, individual responsibilities and the influence of friends, as well as an emphasis on fun without ecstasy, should be considered for inclusion in interventions. The reasoning for non-use and cessation found in this Brazilian sample were similar to USA and Europe studies. Thus, interventions tested in other countries could possibly be adapted to Brazil.

This study highlights the need for multiple preventive level public health interventions in party contexts. For ex-moderate users, environment and social network were among the most important factors for cessation. Harm Reduction is an additional strategy that merits further study in Brazil. The results demonstrate the importance of withdrawal from the electronic scene to support cessation of use. Yet, some respondents suggested that ecstasy consumption is migrating beyond the electronic scene into other party contexts. It is therefore necessary to study and monitor other specific populations in the future.

## Competing interests

Both authors declare that they have no competing interest.

## Authors' contributions

Both authors conceptualized the study, participated in its design and performed the content analysis. MACC conducted the interviews and coding process. Both authors drafted and approved the manuscript.

## Pre-publication history

The pre-publication history for this paper can be accessed here:

http://www.biomedcentral.com/1471-2458/12/353/prepub
